# The association between serum copper concentrations and elevated blood pressure in US children and adolescents: National Health and Nutrition Examination Survey 2011–2016

**DOI:** 10.1186/s12872-021-01880-3

**Published:** 2021-01-28

**Authors:** Changsong Liu, Yanfen Liao, Zongyuan Zhu, Lili Yang, Qin Zhang, Li Li

**Affiliations:** 1 Department of Cardiology, The Third Affiliated Hospital of Chongqing Medical University, Chongqing, 401120 China; 2 Department of Stomatology, The Seventh Affiliated Hospital Sun Yat-Sen University, Shenzhen, 518000 China; 3 Department of Huiqiao Building, Nanfang Hospital, Southern Medical University, Guangzhou, 510515 China; 4 The Third Affiliated Hospital of Chongqing Medical University, 1 Shuanghu Branch Road, Yubei District, Chongqing, China

**Keywords:** Elevated blood pressure, Copper, Dose–response, Children and adolescents

## Abstract

**Background:**

Copper is an essential trace metal with potential interest for cardiovascular effects. Few studies have explored the association between copper and blood pressure in children and adolescents.

**Method:**

We conducted a cross-sectional analysis of 1242 children and adolescents aged 8–17 years who participated in the 2011 to 2016 National Health and Nutrition Examination Survey. Using 2017 American Academy of Pediatrics guidelines, elevated blood pressure (EBP) was defined as a mean systolic and/or diastolic blood pressure (BP) ≥ 90th percentile for sex, age, and height for children aged 1–12 years and systolic BP ≥ 120 mmHg or diastolic BP ≥ 80 mmHg for adolescent age 13–17 years. Mean serum copper was 114.17 μg/dL.

**Results:**

After multiple adjustments, dose–response analyses revealed that EBP was associated with progressively higher serum copper concentrations in a nonlinear trend. In comparison with the lowest quartile of serum copper concentrations, the adjusted odds of EBP for the highest quartile was 5.26 (95% confidence interval, 2.76–10.03).

**Conclusion:**

Our results suggested that high serum copper concentrations were significantly associated with EBP in US children and adolescents.

## Background

Elevated blood pressure (EBP) among children and adolescents has become an important public health challenge in the United States [1]. Recent epidemiological studies have suggested that accumulation of essential trace metals may play a critical role in the development of hypertension [2–4].

Copper is an essential trace metal with antioxidant properties mediated through many redox enzymes [5]. Overload of this metal leads to Fenton-type redox reactions, resulting in oxidative injury [6]. Results of pathological accumulation of copper are observed in Wilson’s disease, a recessively inherited disorder of copper metabolism [7]. Additionally, evidence for the relationship between high copper concentrations and heart failure, Parkinson’s disease, and ischemic stroke were found in epidemiological studies [8–10]. However, evidence regarding the association between serum copper and blood pressure (BP) have been inconsistent or conflicting and most research has been conducted in adult populations [11–14]. A recent study found serum copper to be associated with several cardiovascular disease risk factors in US children and adolescents, including increased total cholesterol, glycohemoglobin and fasting insulin. Nevertheless, no association was found with BP levels [15]. However, this study used BP as a continuous variable and ignored the impact of age, sex and height on the definition of blood pressure level in children and adolescents.

In the current study, using the data from the National Health and Nutrition Examination Survey (NHANES) 2011–2016, and the new 2017 American Academy of Pediatrics guidelines (AAP) [1], we aimed to evaluate the association and dose–response relationship between serum copper and EBP in US children and adolescents.

## Methods

### Study population

NHANES is a nationally representative, multistage survey of the noninstitutionalized US civilian population. All participants provided written informed consent (parental consent was obtained for those < 18 years) and NHANES was approved by the National Center for Health Statistics’ Ethics Review Board. Detailed information on NHANES data collection and survey procedures used in this analysis are publicly available and can be found elsewhere [16].

**Fig. 1:**
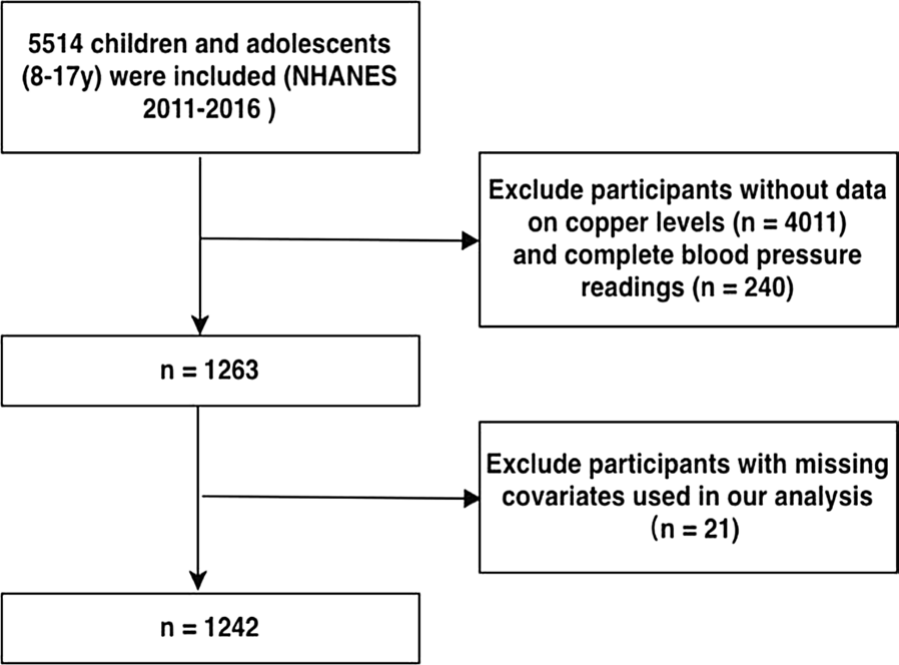
Flow diagram of the selection of eligible participants, National Health and Nutrition Examination Survey 2011–2016

Participants who had completed blood pressure reading and serum copper measure were included in our analyses. Individuals missing important covariates were excluded. The process of data inclusion is presented in Fig. 1.

### Study exposure

Serum specimens were processed, stored at appropriate temperatures (− 70 °C), and shipped to the Division of Laboratory Sciences, National Center for Environmental Health, Centers for Disease Control and Prevention, Atlanta, GA for analysis. Inductively coupled plasma dynamic reaction cell mass spectrometry (ICP-DRC-MS) was used for trace level elemental analysis. The isotopes measured included zinc (m/z 64), copper (m/z 65), and selenium (m/z 78) and the internal standard gallium (m/z 71). Serum samples were diluted with water and a diluent containing gallium (Ga) for multi-internal standardization. The serum to water to gallium dilutant ratio was 1:1:28.

### Definition of EBP

BP measurements were obtained by a certified examiner using an appropriate cuff size and a mercury manometer. Three BP readings were obtained after the participant had rested for 5 min in a seated position with feet flat on the floor. Mean systolic and diastolic BP for each participant were calculated from the recorded readings. Using the 2017 American Academy of Pediatrics guidelines, we classified participants as having EBP or normal BP consistent with previous reports [17]. For children aged 1–13 years, EBP was defined as the mean systolic and/or diastolic BP percentile ≥ 90th percentile for sex, age, and height. For adolescents aged 13–17 years, EBP was defined as SBP at least 120 mmHg and/or DBP at least 80 mmHg.

### Covariates

Demographic information included age, gender, race (Mexican American, other Hispanic non-Hispanic White, non-Hispanic Black, and other race) and family monthly poverty level index category. Other covariates included body mass index (BMI), serum cotinine, physical activity, energy intake and other trace metals (serum selenium, serum zinc, urinary manganese, urinary lead, urinary strontium, urinary arsenic and urinary mercury). BMI was calculated as measured weight in kilograms divided by measured height in meters squared, and BMI percentiles were calculated based on the CDC's BMI-for-age sex-specific growth charts. BMI categories were defined as follows: “underweight”, BMI < 5th percentile; “normal,” BMI 5th to < 85th percentiles; “overweight”, BMI 85th to < 95th percentiles; “obese,” BMI ≥ 95th percentile. Physical activity was assessed by the amount of television, video game and computer usage daily and was classified as high (≤ 2) and low (> 2) in concordance with previous report [18]. Energy intake data were obtained from two 24-h dietary recall interviews and calculated as an average of 2-day energy intake. Serum cotinine, the primary proximal metabolite of nicotine, is generally regarded as the marker of exposure to environmental tobacco smoke.

### Statistical analysis

Differences between groups were tested by the Chi-square test for categorical data and the independent Student t-test, Mann–Whitney-U test, analysis of variance, or the Kruskal–Wallis test for continuous data, as appropriate. Binary logistic regression models were performed to estimate the association between serum copper and EBP. In multivariate logistic regressions, model 1 adjusted for age and sex, model 2 further adjusted for race, family monthly poverty level category, physical activity, BMI, total energy intake, serum cotinine, and other trace metals. The following sensitivity analyses were performed: (1) Multivariable linear regression was used to examine whether the associations between serum copper and BP were significant when SBP and DBP were analyzed as continuous variables (2) Analyses were performed with the sample stratified into children (8–12 years) and adolescents (13–17 years). We assessed for collinearity between adjustment variables by calculating variance inflation factors. The dose–response relationship was conducted by restricted cubic spline with three knots. We performed tests for linear trend by entering the median value of each category of copper as a continuous variable in the models. All analyses were performed using Stata 15.1 and R 3.3.0 software. All reported probabilities (*p*-values) were two-sided with *p* < 0.05 considered as significant.

## Results

Overall, the study cohort included a total of 1242 US children and adolescents aged 8–17 years at baseline. Table 1 presents the characteristics of the study participants. The mean serum copper concentrations was 114.17 μg/dL. The overall prevalence of EBP was 14.0%. EBP was more likely to occur in males (132 versus 42, *P* < 0.01) and in Non-Hispanic Black participants (*P* < 0.01). Participants with EBP tend to be older (12.9 versus 12.0 years, *P* = 0.01), overweight and obese (*P* < 0.01), consumed more calories (2152 versus 1878 kcal, *P* < 0.01) and had lower amount of physical activity (*P* = 0.01) compared to those without EBP. There was no difference in serum copper concentrations between genders (male versus female, 114.3 versus 114.1 μg/dL, *P* = 0.89). Non-Hispanic blacks had higher mean serum copper concentrations compared to non-Hispanic whites, Mexican-Americans and other Hispanic (122.4, 112.1, 110.9 and 109.5 μg/dL, respectively, *P* < 0.01). Mean serum copper concentrations were higher in participants with EBP compared to those without EBP (125.5 versus 112.3 μg/dL, *P* < 0.01).

**Table 1:** Characteristics of the study population by serum copper quartile

	Total (n = 1242)	1^st^ (n = 312)	2^nd^ (n = 309)	3^rd^ (n = 312)	4^th^ (n = 309)	*P* value
Male	624 (50.2)	168 (53.8)	135 (43.7)	138 (44.2)	183 (59.2)	< 0.001
Race/ethnicity						< 0.001
Mexican American	261 (21.0)	60 (19.2)	81 (26.2)	66 (21.2)	54 (17.5)	
Other Hispanic	120 (9.7)	30 (9.6)	33 (10.7)	39 (12.5)	18 (5.8)	
Non-Hispanic White	300 (24.2)	78 (25.0)	87 (28.2)	72 (23.1)	63 (20.4)	
Non-Hispanic Black	357 (28.7)	75 (24.0)	63 (20.4)	81 (26.0)	138 (44.7)	
Other Race	204 (16.4)	69 (22.1)	45 (14.6)	54 (17.3)	36 (11.7)	
Family monthly poverty level category						0.001
< 1.30	579 (46.6)	144 (46.2)	123 (39.8)	138 (44.2)	174 (56.3)	
1.31–1.85	174 (14.0)	57 (18.3)	51 (16.5)	39 (12.5)	27 (8.7)	
> 1.85	438 (35.3)	105 (33.7)	126 (40.8)	117 (37.5)	90 (29.1)	
Age (years)	12.2 (2.9)	13.5 (2.5)	12.2 (2.9)	11.7 (3.0)	11.3 (2.9)	< 0.001
BMI						< 0.001
Underweight	39 (3.1)	12 (3.8)	15 (4.9)	3 (1.0)	9 (2.9)	
Normal	714 (57.5)	231 (74.0)	183 (59.2)	174 (55.8)	126 (40.8)	
Overweight	225 (12.5)	39 (21.4)	66 (22.1)	69 (16.5)	51 (18.1)	
Obese	264 (21.3)	30 (9.6)	45 (14.6)	66 (21.2)	123 (39.8)	
Height, cm	152.1 (15.6)	153.1 (13.6)	152.4 (14.8)	150.9 (14.2)	152.0 (15.6)	0.243
Energy intake (kcal/day)	1920 (635)	2000 (681)	1900 (661)	1870 (559)	1890 (630)	0.063
Physical activity						0.154
Low	558 (44.9)	132 (42.3)	135 (43.7)	135 (43.3)	156 (50.5)	
High	684 (55.1)	180 (57.7)	174 (56.3)	177 (56.7)	153 (49.5)	
SBP, mm Hg	105.1 (9.4)	103.2 (8.7)	104.0 (8.7)	105.1 (10.0)	108.0 (9.6)	< 0.001
DBP, mm Hg	62.6 (6.1)	59.3 (4.8)	61.8 (6.4)	63.8 (6.6)	65.6 (4.6)	< 0.001
Serum copper, μg/dL	114.2 (26.5)	86.7 (7.7)	103.2 (4.2)	118.3 (5.2)	148.7 (25.0)	< 0.001
Serum cotinine (ng/mL)	3.8 (30.1)	10.3 (56.0)	3.0 (19.2)	0.9 (6.9)	0.9 (2.3)	0.006
Urinary manganese (μg/L)	0.2 (0.19)	0.19 (0.16)	0.17 (0.12)	0.19 (0.28)	0.19 (0.19)	0.347
Urinary lead (μg/L)	0.47 (0.5)	0.42 (0.3)	0.40 (0.3)	0.44 (0.4)	0.60 (0.8)	0.001
Urinary strontium (μg/L)	131 (123)	144 (117)	119 (100)	142 (167)	118 (89)	0.003
Urinary mercury (μg/L)	0.49 (0.71)	0.50 (0.74)	0.49 (0.72)	0.44 (0.70)	0.54 (0.67)	0.339
Urinary arsenic (μg/L)	11.3 (26.4)	13.5 (44.5)	9.8 (12.8)	12.0 (16.7)	10.1 (18.9)	0.273
Serum selenium (μg/L)	121 (16.6)	122 (13.3)	120 (13.8)	121 (13.7)	120 (23.4)	0.202
Serum zinc (μg/dL)	83.7 (16.3)	80.9 (12.7)	82.2 (14.1)	84.4 (13.4)	87.4 (22.5)	< 0.001

BMI, body mass index; SBP, systolic blood pressure; DBP, diastolic blood pressure

**Fig. 2:**
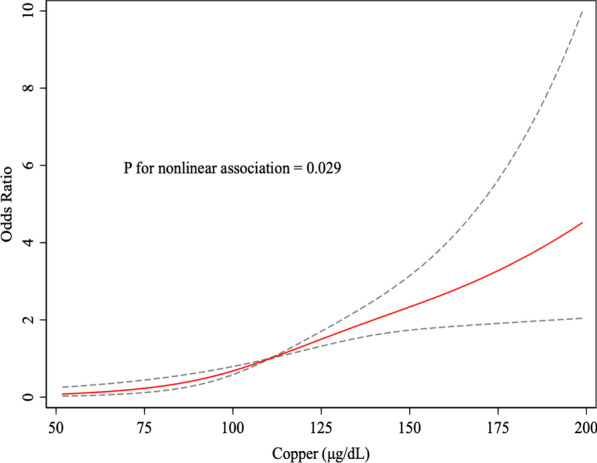
Dose–response relationships between serum copper and elevated blood pressure. The associations were adjusted for age, gender, race (Mexican American, other Hispanic non-Hispanic White, non-Hispanic Black, and other race) and Family monthly poverty level index category, BMI, serum cotinine, physical activity, energy intake and other trace metals. The solid line and dashed line represent the estimated OR and its 95% CI. BMI, Body Mass Index; OR, odds ratio; CI, confidence intervals

Table 2 presents the odds ratios of EBP based on quartiles of serum copper concentrations. In crude logistic regression analyses, high levels of serum copper were significantly associated with increased risk of EBP. After adjustment for age and sex (model 1), serum copper concentrations had a significantly positive association with EBP. After further adjustment for race, family monthly poverty level category, physical activity, BMI, total energy intake, serum cotinine, and other trace metals (model 2), the results remained stable and statistically significant. When BP was analyzed continuously, higher serum copper (one-SD increase) was associated with both higher SBP (1.87 [1.36–2.38] < 0.001) and DBP (2.19 [1.83–2.55] < 0.001) levels. Results stratified by children and adolescents were both consistent with the main analysis. Dose–response relationship between serum copper and EBP were shown in Fig. 2. In restricted cubic spline model, excessive serum copper increased the risk of EBP in a linear manner (*P* = 0.029).

**Table 2:** Weighted odds ratios and 95% confidence intervals for elevated blood pressure according to quartiles of serum copper

Serum copper (μg/dL)	Events/cases	OR(95%CI)		
		Crude	Model1^a^	Model 2^b^
Quartile 1	27/312	Ref	Ref	Ref
Quartile 2	30/279	1.14 (0.66–1.96)	1.87 (1.05–3.32) *	1.80 (0.94–3.43)
Quartile 3	45/267	1.78 (1.07–2.95) *	3.37 (1.94–5.85) **	3.70 (1.97–6.95) **
Quartile 4	72/237	3.21 (2.00–5.16) **	5.44 (3.21–9.22) **	5.26 (2.76–10.03) **
P for trend	NA	< 0.001	< 0.001	< 0.001
Per 1 SD serum copper		1.52 (1.32–1.75) **	1.76 (1.50–2.06) **	1.69 (1.38–2.07)**

CI, confidence interval; OR, odds ratio; NA, not applicable; SD, standard deviation

^*^P < 0.05; **P < 0.01

^a^Model 1 were adjusted by age and sex

^b^Model 2 were adjusted by age, sex, race, family monthly poverty level category, physical activity, body mass index, total energy intake, serum cotinine, and other trace metals

## Discussion

In this study, using a nationally representative large-scale database and updated classification system for pediatric BP, we found that serum copper was associated with increased risk of EBP in the US population aged between 8 and 17 years old. The association was statistically significant despite adjustments for the impact of co-exposure to multiple metals [19, 20].

In animal experiments, plasma copper concentrations were significantly higher in hypertensive rats [21]. Other animal studies have demonstrated implanting a copper cuff results in neointimal thickening in response to vascular injury [22], while copper chelators inhibit the development of vascular inflammation and new intima formation in response to vascular injury [23, 24]. In humans, evidence linking serum copper and blood pressure has been inconsistent. Most research conducted thus far has been in adults. Taneja et al. [25]. conducted a study with 500 adult participants which noted higher levels of copper in urine in hypertensive subjects (2.70 ± 0.10 versus 5.14 ± 0.15 mg/dL). Conversely, Li et al. [26]. found no difference. Additionally, Kim et al. [27]. found that copper intake was inversely associated with SBP and DBP in 258 adult subjects.^.^ There is a dearth of studies investigating the association between copper and blood pressure in children and adolescents. In a cross-sectional study conducted among 1427 US children and adolescents, no association was found between serum copper concentrations and blood pressure[15]. Another study among European children suggested a positive association between serum copper and diastolic blood pressure [28]. However, a primary deficit of these studies was that they did not use blood pressure percentiles to define hypertension, which is the current standard for delineating normotension from hypertension in children and adolescents. Using the 2017 hypertension diagnostic criteria, our results indicate that high serum copper concentrations increase the risk of EBP in children and adolescents. Previous studies among adult populations have yielded inconsistent results, with some studies affirming a role of Copper in EBP and others suggesting the converse. It should be noted that the pediatric sample included in this study may not be directly comparable to research performed on adult samples. First, there is a dearth of longitudinal clinical trials on hypertension into adulthood. In fact, lack of such data has led to a grade of incomplete regarding BP evaluation in childhood by the United States Preventive Services Task Force [29]. Therefore, it is difficult establish whether the contribution of risk factors such as copper levels is independent of the age of onset of hypertension. Additionally, different standards in the definition of EBP in adults and children may have resulted in different results.

The mechanisms behind the association between serum copper and BP remain unclear. It has been reported that Reactive oxygen species (ROS) disrupts the balance of oxidation and antioxidant systems. Excessive copper can affect the activity of ROS [5]. The resultant increase in superoxide may lead to the impairment and dysfunction of endothelial structure, and consequently result in the development of EBP [30]. Furthermore, high copper concentrations can reduce myosin-ATPase activity, resulting in calcium overload [31]. Increased Ca^2+^ in smooth muscular layer of vessels may cause an increase in the arterial wall tension finally resulting in EBP [32].

As suggested by previous literature, serum copper appears to reflect the status of copper nutrition in both depleted and replete populations. Pediatric reference intervals for serum copper concentrations are not well established [33]. Only a few studies have measured serum copper concentrations in children and adolescents [33]. The reference values of serum copper concentrations in US were higher than the counterparts in Mexico and Egypt and was the same as in China. In our study, the average serum copper concentrations in participants with EBP was 125.5 ± 32.8 μg/dL and in those with normal BP was 112.3 ± 24.2 μg/dL, which were within the US reference range. We provide the first evidence of a correlation between serum copper and EBP in US children and adolescents.

Our study has several strengths. This is the first study to explore the relationship between serum copper and EBP since the standards for pediatric blood pressure were updated in 2017. In addition, we investigated the dose–response relationship between serum copper and EBP. Furthermore, we considered the simultaneous impacts of various metals in our analyses to capture the complex nature of environmental exposure as a whole.

However, this study also has some limitations. First, this is a cross-sectional study, and it is difficult for us to determine causality. Second, although a number of potential confounders were controlled, we cannot rule out the possibility of unmeasured confounding factors. Third, the complicated interactions of many trace metals with copper warrants further investigation.

## Conclusions

In conclusion, our study has found a positive and non-linear association between serum copper and elevated blood pressure in US children and adolescents. We hope that this study can help policy makers develop safer reference ranges in US children and adolescents. Further prospective studies are needed to confirm our results and elucidate the mechanisms involved.

## Data Availability

Anonymized data are available from the National Center for Health Statistics (https://www.cdc.gov/nchs/nhanes/about_nhanes.htm) or from the corresponding author on reasonable request.

## Abbreviations

EBP: Elevated blood pressure
BP: Blood pressure
NHANES: National Health and Nutrition Examination Survey
AAP: American Academy of Pediatrics guidelines
ICP-DRC-MS: Inductively coupled plasma dynamic reaction cell mass spectrometry
Ga: Gallium
BMI: Body mass index
ROS: Reactive oxygen species
